# Translation, adaptation and testing of an emergency care satisfaction scale in Swedish pediatric emergency departments

**DOI:** 10.1186/s12887-021-02961-0

**Published:** 2021-11-03

**Authors:** Anne Wennick, Dorota Schoug, Anna Ekwall, Malin Axelsson

**Affiliations:** 1 Department of Care Science, Faculty of Health and Society, Malmö University, Jan Waldenströms gata 25, 205 06 Malmö, Sweden; 2 Department of Pediatrics, Skåne University Hospital, Lund, Sweden; 3 Department of Health Sciences, Faculty of Medicine, Lund University, Lund, Sweden

**Keywords:** CECSS, Children, Emergency departments, Reliability, Satisfaction, Translation, Validity

## Abstract

**Background:**

Pediatric healthcare today shows a rising demand for research focusing on children’s perspectives on and consumer satisfaction with the nursing care they receive. Therefore, the purpose of this study was to translate and adapt the Consumer Emergency Care Satisfaction Scale (CECSS), a paper-based, self-administered 19-item questionnaire originally developed in the United States and targeted towards adults, and then test the new version in Swedish pediatric emergency departments.

**Methods:**

The study was designed with a two-phase approach. Firstly, a forward–backward translation of the CECSS, involving expert consensus, was performed, and then the questionnaire was adapted for children aged 10–18 and assessed for face and content validity. Secondly, the translated and adapted questionnaire was tested with a clinical sample for construct validity, internal consistency, and reliability. This last aspect was assessed using a structured telephone interview 7–10 days after the participant visited a pediatric emergency department. All children participating in this study gave their assent (< 15 years) or consent (≥ 15 years), and their guardian’s written informed consent was also obtained.

**Results:**

The paper-based, self-administered 19-item Swedish version of the CECSS was tested on a clinical sample consisting of 203 nonurgent children (boys: n = 109, 53.7 % and girls: n = 94, 46.3 %) between 10 and 18 years (mean age 13.8, SD 2.29). The factor analysis revealed three factors that explain 63.1 % of the total variation in the 15 items. The Cronbach’s alphas for the three dimensions (*caring*, *teaching*, and *clinical competence*) varied between 0.79 and 0.88. The intraclass correlation coefficient (ICC) for the entire Swedish version of the CECSS was 0.58, and the ICCs for the three dimensions varied between 0.56 and 0.71.

**Conclusions:**

The results show that the developed Swedish Pediatric Consumer Emergency Care Satisfaction Scale (p-CECSS-S) is a valid, stable and easy-to-use-questionnaire that can be used to assess children’s satisfaction with nursing care.

## Background

Satisfaction is a hard-to-measure concept that continues to challenge researchers today [1–8]. However, when satisfaction is used as a quality indicator of, for instance, a person’s nursing care, it is reasonable to conclude that the best source of information is the person who has received the care. Historically, in pediatric care, healthcare professionals have mainly communicated about children’s health care matters with the child’s legal guardian [4, 7, 8] using a supposed child perspective. This perspective is, in fact, only an adult’s outside perspective of the child’s condition, experiences, perceptions and actions, though with the child’s best interests in mind [9]. However, it is not always an accurate picture of the child’s situation [10]. Therefore, there is currently a demand for research that focuses not only on children’s perspectives but also on their satisfaction as the recipient of nursing care in a pediatric healthcare setting. With this in mind, this study was designed to test a questionnaire developed to measure children’s satisfaction with nursing care in Swedish pediatric emergency departments (EDs).

Several attempts have been made to develop scales that measure satisfaction with nursing care in different settings [11–15]. In 1975, Risser developed a scale to measure satisfaction with nursing care in a primary care setting. Subsequently, based on Risser’s work, Davis and Bush [14] developed a scale specifically for EDs – the Consumer Emergency Care Satisfaction Scale (CECSS). The original CECSS is a standardized questionnaire consisting of 19 statements divided into two dimensions, *caring* and *teaching* [16], graded on a 5-point Likert scale from 1 (completely disagree) to 5 (completely agree) [14]. In the questionnaire, the caring dimension consists of 12 statements concerning nurses’ involvement in a person’s nursing care, and the teaching dimension contains three statements related to information or education on the person’s individual condition. The four remaining statements are inversely worded to reduce response bias [14].

The CECSS questionnaire was initially developed in the United States [14, 16] but has subsequently been used internationally [17–25]. It was translated into Swedish by Ekwall and Davis [26] and then tested on persons aged 18–91 years at an adult ED and in two adult care units. This resulted in a modified Swedish version of the CECSS. Since then, this modified version has been used in a study with persons aged 22–87 arriving at an adult ED by ambulance [27]. The use of the CECSS with children was described in a study that aimed to measure children’s satisfaction with triage care [28]. El-Khedr and El-Gawad [28] report the Cronbach’s alpha value but not how the questionnaire was validated to the context or respondents. Although previous research has shown that the CECSS has satisfactory psychometric properties [14, 29], currently, no age-adapted translated version has been tested in pediatric Eds. Hence, the aim of this study was to translate, adapt and test the CECSS in Swedish pediatric EDs. As a result, Dr. Barbara Davis, a professor of nursing who has the rights to the CECSS, was contacted to obtain permission to psychometrically test the questionnaire at Swedish healthcare clinics for children (e.g., pediatric EDs).

## Methods

### Design

The study was designed with a two-phase approach. Firstly, to achieve consistency, the original CECSS was subjected to a translation process comprising the five guiding steps described by Beaton et al. [30]. This started with a forward–backward translation of the original paper-based, self-administered 19-item CECSS, whose purpose is to measure satisfaction with nursing care [14]. In addition, the translated questionnaire was tested for face and content validity with an expert panel of pediatric nurses (PNs) and another panel of children. Secondly, the translated and adapted questionnaire was tested using classical test theory as described by Streiner et al. [31] on a clinical sample at two Swedish pediatric EDs; construct validity (factor analysis), internal consistency (Cronbach’s alpha), and reliability according to the test–retest method were assessed. The last assessment was performed as a structured telephone interview 7–10 days after having visited the pediatric ED. The rational for performing the abovementioned psychometric analysis was to mimic the analyses used in previous studies involving the CECSS (e.g., [14, 16, 32]). The annual volume at the target pediatric EDs is approximately 18,500 children aged 0–18 years, and during the data-collection period, these Eds were visited by about 8,000 children. Most of the children were younger than the lower inclusion age (10 years) set in this study. The regional ethical review board in Lund, Sweden, approved this study (Reg. No. 2016/310).

### Forward–backward translation

The translation process followed the forward–backward translation steps described by Beaton et al. [30] and started with two authorized independent translators performing a forward translation into Swedish. One translator had knowledge of pediatric EDs and nursing care. The translations were subsequently compared by the current researchers, and after consensus was reached, a synthesized Swedish version was obtained. Thereafter, two more independent translators whose mother tongue was English, the original language of the CECSS, but who had no specific knowledge of the subject performed a backward translation to English. Once again, the translations were compared by the researchers, and after a consensus was reached, a synthesized English version was obtained. This version was then crosschecked by the researchers for accuracy against the original CECSS.

### Face validity and content validity with pediatric nurses

To test the Swedish version of the CECSS for face and content validity, a representatively selected expert panel was recruited to index the relevance of each item using the *item-content validity index* (I-CVI) and each questionnaire item using the *scale-content validity index* (S-CVI) as described by Lynn [32]. The recruitment was performed on the basis of a targeted selection with a predetermined criterion: an even distribution of PNs operating at one of four pediatric EDs in southern Sweden. This resulted in a total of seven experts who gave their written informed consent to participate. This number (*n* = 7) was determined to be sufficient, given that Lynn [32] recommends that a panel consist of at least three and no more than ten experts. Yet another recommendation advocated by Lynn [32] is that the indexing be based on a 4-degree ordinal scale without any given midpoint, as it forces all experts to take a stand for their assertions in a positive or negative direction, enabling dichotomization. For this reason, the I-CVI and S-CVI were graded from 1 = not relevant, 2 = quite relevant, 3 = relevant and 4 = highly relevant.

### Language understanding with children from a scout corps

To determine whether the content of the Swedish version of the CECSS was understandable to children aged 10–18 years, children in this age group who could read and understand Swedish were recruited via a leader at a local scout corps using convenience sampling for a think-aloud interview as described by Fonteyn et al. [33]. The lower age limit was set at 10 years after the questionnaire was independently piloted with three ten-year-olds with varied reading ability.

All target children were given detailed, age-appropriate information about the study orally by the second author and in writing addressing both the child and the child’s guardian. After having been given time to reflect, seven children gave their assent (< 15 years) or consent (≥ 15 years), and their guardian’s written informed consent was provided as well. Thereafter, a time and place for the think-aloud interview were selected according to the wishes of the child and her or his guardian. With their permission, the interviews were audio-recorded for later verbatim transcription. The think-aloud interview was performed by the second author, a PN with several years of experience in the profession who is well versed in communicating with children.

### Construct validity and internal consistency with a clinical sample

To test the Swedish version of the CECSS for construct validity and internal consistency, a clinical sample of children seeking pediatric emergency care at two departments in southern Sweden were asked to participate in the current study by an experienced PN (at the respective department) who was engaged as a gatekeeper but was not involved in the child’s care. The inclusion criteria for participation were checked by the gatekeeper and were as follows: children aged 10–18 who could read and understand the Swedish language and who were triaged to nonurgent categories (non-life-threatening but necessitates emergency medical care within a reasonable time; non-life-threatening but necessitates care within a reasonable time) according to the 4-level Rapid Emergency Triage and Treatment System Pediatrics (RETTS-p) algorithm. Exclusion criteria were children who, according to the RETTS-p algorithm, were triaged to rapid (< 20 min; potentially life-threatening) or immediate care (life-threatening) and whose condition did not improve during their stay at the ED.

**Fig. 1:**
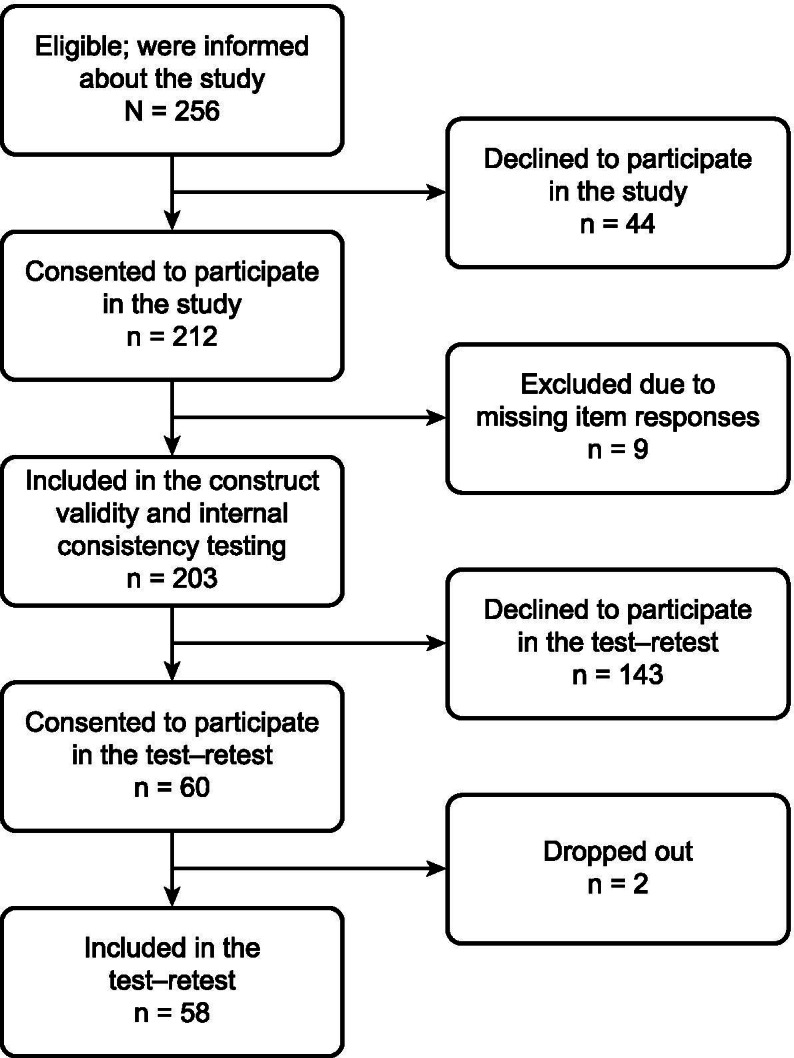
An overview of the study sample included in the testing for construct validity and internal consistency and in the test–retest

To emphasize that this study was not part of the child’s care, those deemed eligible were only approached once the child was being discharged from the pediatric ED. Approaching them upon discharge meant that the participants and their guardians could feel confident that they could withdraw from the study at any time without any detriment to the child’s care. In addition, they were informed that participation was voluntary. In total, 256 potential participants were given detailed, age-appropriate information about the study, orally by the second author and in writing addressing the child and the child’s guardian. Ultimately, 212 children gave their assent or consent, depending on their age, along with their guardian’s written informed consent and answered the questionnaire. However, nine questionnaires had to be excluded due to missing item responses. The sampling procedure is described in Fig. 1. The data was analyzed using explorative factor analysis and Cronbach’s alpha coefficient in the Statistical Package for the Social Sciences (SPSS; IBM SPSS Statistics 23, IBM, New York, US).

### Test–retest with a clinical sample

Of the 203 children who completed the Swedish version of the CECSS to assess construct validity and internal consistency, 60 gave their assent (age < 15) or consent (age ≥ 15) along with their guardian’s written informed consent to answer the questionnaire by telephone. However, two dropped out, resulting in a sample of 58 children. Again, the sampling procedure is depicted in Fig. 1.

## Results

### Testing of face validity and content validity with pediatric nurses

Content validity was tested by an expert panel consisting of seven PNs with 4–23 years of experience working in Swedish pediatric EDs. Their I-CVIs varied between 0.43 and 1.00; the I-CVI calculations for each of the 19 items in the Swedish version of the CECSS are presented in Table 1. In summary, seven of the 19 items had a rating lower than 0.8. Of the seven items with a rating lower than 0.8, four (5, 9, 14, 17) were negatively worded to minimize the risk of response bias, and the remaining three (3, 7, 15) focused on the nurse’s professional practice. After some minor linguistic editing, all 19 items were included in the Swedish version of the CECSS.

**Table 1:** Content validity assessed by the expert panel *n* = 7 (S-CVI/Ave: 0.83)

Item	I-CVI
1	0.86
2	0.86
3	0.57
4	0.86
5	0.57
6	1.00
7	0.57
8	1.00
9	0.71
10	1.00
11	1.00
12	1.00
13	1.00
14	0.43
15	0.71
16	1.00
17	0.57
18	1.00
19	1.00

### Testing of language understanding with children from a scout corps

The language clarity of the paper-based, self-administered 19-item Swedish version of the CECSS was tested by a think-aloud panel consisting of boys (n = 5) and girls (n = 2) aged 10 (n = 2), 12 (n = 1), 13 (n = 1), 14 (n = 1), 15.5 (n = 1) and 17.5 (n = 1). Individually, all seven children read the 19 items aloud one at a time and verbally described what they understood each to mean.

The subsequent analysis of their interviews showed that the children had difficulty understanding certain expressions and words in the Swedish version. For example, Item 6, *“The nurse talked about what problems I should be aware of”*, and Item 7, *“The nurse talked about what could happen once I was back home”*, were difficult to interpret and were understood to be asking a similar question. In addition, Item 11, *“The nurse was understanding when I told him or her about my problems”*, was interpreted to mean that the nurse understood the child’s story, not that the nurse was empathetic. Lastly, Item 14, *“The nurse treated me like a number and not as a person”*, was clearly difficult to interpret. In addition to this, the children had difficulty understanding the following words used in the items: *skill*, *action*, *treatment*, *attentiveness* and *care.*

Ultimately, the expressions and words mentioned above caused language difficulties and were discussed with four special educators with experience working with children in pediatric departments. Based on their suggestions, the wording was adjusted to be more child-friendly but with the same meaning. Moreover, the synonyms suggested for words that the children had described as difficult to understand were then used to further optimize the language understanding among children.

### Testing of construct validity on a clinical sample

The paper-based, self-administered 19-item Swedish version of the CECSS was tested for construct validity on a clinical sample consisting of 203 nonurgent children (boys: n = 109, 53.7 %; girls: n = 94, 46.3 %) between 10 and 18 years old (mean age 13.8, SD 2.29). In the first step, two factor analyses were performed with two default factors in accordance with the original CECSS, which has two dimensions. In the first factor analysis, the Swedish version (including 19 items) was tested. This factor analysis explained 44.3 % of the total variation. The negatively worded items, numbers 5, 9, 14 and 17, had loadings lower than < 0.3 and were consequently excluded from the second factor analysis.

The second factor analysis performed with 15 items (1, 2, 3, 4, 6, 7, 8, 10, 11, 12, 13, 15, 16, 18, 19) explained 54.7 % of the total variation. Of the 15 items, ten items loaded on factor one (1, 8, 10, 11, 12, 13, 15, 16, 18, 19), and five items loaded on factor two (2, 3, 4, 6, 7). The first factor was *caring* and the second *teaching*. The alpha coefficient values ​​for the entire Swedish version of the CECSS with 15 items was 0.87, with *caring* reaching 0.88 and *teaching* reaching 0.85.

In the second step, two factor analyses with the 19 items of the Swedish version were performed. The first analysis was performed without any default number of factors. The correlation matrix showed that the 19 items were correlated. The factor analysis identified five factors that accounted for 63.4 % of the total variation. Specifically, ten items loaded on the first factor, three loaded on the second, two loaded on the third, two loaded on the fourth, and two loaded on the fifth.

After reviewing the loadings and the scree plot, three factors could clearly be considered most relevant for the Swedish version of the CECSS. Subsequently, a second factor analysis was performed with a predetermined number of factors with the highest eigenvalues (three factors). The three factors explained 51.7 % of the total variation. The three factors, factor loadings and eigenvalues ​​are presented in Table 2.

**Table 2:** Factors and factor loadings in the factor analysis of the Swedish version of the CECSS, including negatively worded items

Item number	Factor 1	Factor 2	Factor 3	Cronbach’s alpha
1	0.652			
3	0.518			
8	0.509			
10	0.739			
11	0.789			
12	0.727			
13	0.554			
15	0.771			
16	0.525			
17	0.307^a^			
18	0.801			
19	0.776			0.87
2		0.493		
4		0.851		
6		0.810		
7		0.830		
5		0.339^a^		0.76
9			0.501^a^	
14			0.712^a^	0.35
Entire scale				0.86

^a^ negatively worded item

### Testing of internal consistency on a clinical sample

The internal consistency of the paper-based, self-administered 19-item Swedish version of the CECSS was tested with Cronbach’s alpha; the alpha values for the entire Swedish version and for each factor are presented in Table 3. In connection with the alpha coefficient calculation, the alpha values were checked with certain items excluded. For example, in factor 1, the alpha would be 0.88 if Item 17 were excluded. In factor 2, the alpha would be 0.80 if Item 5 were excluded. In factor three, Items 9 and 14 should be excluded to improve the internal consistency of the Swedish version to 0.87. Therefore, in the final factor analysis, the negatively worded items (5, 9, 14 and 17) were removed.

**Table 3:** Factor loadings and internal consistency of the Swedish version of the CECSS without the negatively worded items (5, 9, 14, 17)

Item number	Factor 1	Factor 2	Factor 3	Variance %	Communalities	Cronbach’s alpha
1	0.565				0.428	
8	0.664				0.541	
10	0.712				0.542	
11	0.700				0.629	
12	0.750				0.613	
13	0.728				0.576	
15	0.649				0.672	
16	0.541				0.342	
18	0.764				0.672	
19	0.756			41.7	0.645	0.88
4		0.827			0.744	
6		0.824			0.755	
7		0.876		13.1	0.793	0.85
2			0.846		0.794	
3			0.763	8.3	0.713	0.79
Entire scale				63.1		0.87

The final factor analysis resulted in a Kaiser–Meyer–Olkin measure (KMO) of 0.88 and showed that 15 items were positively correlated. The factor analysis generated three factors explaining 63.1 % of the total variation in the 15 items. The eigenvalue of these three empirical factors were each > 1. Hence, the final Swedish version of the CECSS tested in pediatric EDs had three dimensions. Based on the content of the items, the dimensions were named *caring*, *teaching*, and *clinical competence*. The internal consistency was calculated again; the Cronbach’s alphas and the three factors are presented in Table 3. The testing was performed using the same clinical sample (n = 203) used for testing the construct validity.

### Test–retest on a clinical sample

On two different measurement occasions 7–10 days apart, repeated measurements were performed with fifty-eight participants from the same clinical sample (n = 203) used to test the construct validity and internal consistency. As presented in Table 4, the intraclass correlation coefficient (ICC) for the entire Swedish version was 0.58, and the ICCs for the three dimensions varied between 0.56 and 0.71. Lastly, the Swedish version was named p-CECSS-S (Table 5). In this case, p- stands for pediatric and -S stands for Swedish.

**Table 4:** Intraclass correlation coefficient (ICC) of the test–retest for the entire questionnaire and each dimension with a 95 % confidence interval (CI)

	ICC	95 % CI
Swedish version of the CECSS	0.58	0.28–0.76
**Dimensions**:		
Caring	0.56	0.25–0.74
Teaching	0.69	0.45–0.82
Clinical competence	0.71	0.51–0.82

**Table 5:** CECSS and p-CECSS-S items by dimension

Item number	Items	Dimension CECSS (p-CECSS-S)
1	The nurse performed his/her duties with skill.	Caring (Caring)
2	The nurse seemed to know something about my illness/problem.	Caring (Clinical competence)
3	The nurse knew what treatment I needed.	Caring (Clinical competence)
4	The nurse gave me instructions about caring for myself at home.	Teaching (Teaching)
5	The nurse should have been more attentive than he/she was.	Negatively worded (Negatively worded)
6	The nurse told me what problems to watch for.	Teaching (Teaching)
7	The nurse told me what to expect at home.	Teaching (Teaching)
8	The nurse explained all procedures before they were done.	Caring (Caring)
9	The nurse seemed too busy at the nurses station to spend time talking with me.	Negatively worded (Negatively worded)
10	The nurse explained things in terms I could understand.	Caring (Caring)
11	The nurse was understanding when listening to my problem.	Caring (Caring)
12	The nurse seemed genuinely concerned about my pain, fear, and anxiety.	Caring (Caring)
13	The nurse was as gentle as he/she could be when performing painful procedures.	Caring (Caring)
14	The nurse treated me as a number instead of as a person.	Negatively worded (Negatively worded)
15	The nurse seemed to understand how I felt.	Caring (Caring)
16	The nurse gave me a chance to ask questions.	Caring (Caring)
17	The nurse was not very friendly.	Negatively worded (Negatively worded)
18	The nurse appeared to take time to meet my needs.	Caring (Caring)
19	The nurse made sure that all my questions were answered.	Caring (Caring)

## Discussion

In this study, the English paper-based self-administrated CECSS questionnaire was forward–backward translated to a Swedish version adapted for children aged 10–18. It was then evaluated for use with its target population in Swedish pediatric EDs. The results demonstrate that the Swedish version (named the p-CECSS-S) was both valid and reliable, which indicates that it can be used to measure children’s satisfaction with nonurgent pediatric emergency care.

The rationale for deciding on a lower age limit than El-Khedr and El-Gawad [28] when testing the CECSS with children aged 12–18 living in Egypt was that the self-reported questionnaire KIDSCREEN-52 was found to be applicable to children aged 8–18 [34]. Therefore, we set the lower age limit between the two after having independently piloted the Swedish version with three 10-year-olds with varied reading ability. If testing the questionnaire with children younger than 10, a smiley Likert scale might be preferable, especially as smileys have been found appropriate for use with the KIDSCREEN-37 with children aged 4–7 [34].

In contrast to the original two-dimension CECSS questionnaire, the p-CECSS-S reached a three-factor solution that contains not only the dimensions of *caring* and *teaching* but also that of *clinical competence*. This is similar to Kristensson and Ekwall’s [35] study, which tested their modified version of the CECSS on the persons accompanying patients in EDs. In the current study and in Kristensson and Ekwall’s [35] study, the dimension of *clinical competence* contained Items 2 and 3 used here. In the latter study, however, a third item was identified, Item 1. All three items (1, 2, 3) derive from the 12-item dimension of *caring* in the original CECSS. Kristensson and Ekwall [35] conclude that the items concern the nurses’ clinical competence, which is in line with our conclusion about why the expert panel in the current study rated Item 3, *“The nurse knew what treatment I needed”*, lower than 0.8. This item may have been found extraneous in a questionnaire supposedly screening for satisfaction with the care provided by the nurse, as it could instead be perceived as screening for satisfaction with the nurse him or herself. Items 3, 7, and 15 were rated lower than 0.8 by the expert panel. Item 7 in the dimension of *teaching*, *“The nurse told me what to expect at home”*, and Item 15 in the dimension of *caring*, *“The nurse seemed to understand how I felt”*, do, however, require another explanation. Consequently, it would have been interesting to specifically ask the expert panel why these items (3, 7, 15) were rated so low. The panel also rated the four negatively worded items (5, 9, 14, 17) low, but that was somewhat expected.

A strength of the development of the p-CECSS-S was that prior to psychometric testing, the questionnaire was translated thoroughly, adhering to the translation procedure described by Beaton et al. [30]. Moreover, the translated version was refined based on the feedback generated during the think-aloud interviews (described by Fonteyn et al. [33]) with the target population, that is, children between 10 and 18 years. In line with the children’s feedback, the questionnaire was slightly refined and then backtranslated into English for comparison with the original version, which showed consistency between the two versions.

One limitation worth discussing is that each item focuses on the nurse, and from our experience, it might be difficult for both children and adults to distinguish between the different professions in a healthcare setting. In other words, when interpreting the results of a screening, one should focus on children’s satisfaction with the care at the target department rather than on the professional providing that care. In addition, whether a sample size of n = 203 respondents offers adequate statistical power for data analysis is open to debate. It is recommended that the sample size be greater than the number of items, which was n = 19 in the current study. Since 10:1 is a widely accepted ratio [36, 37] and a factor analysis sample of n = 200 is considered fair [38–40], the sample size in the current study may be considered adequate. The same criticism might apply to the test–retest sample, as that size was also greater than the number of items (n = 58 and n = 19, respectively). However, if aiming for a test–retest sample with a ratio of 10:1, recruitment must continue until repeated measurements have been performed with 190 different children. In this study, this would have implied the recruitment of a significantly larger clinical sample than the one actually selected, which might be statistically motivated but is perhaps not ethical. This is because of the inconvenience of having to extend the children’s visit to the pediatric ED when already not feeling well just to participate in a study aiming to reach a 10:1 test–retest ratio. Thus, in this study, a sample of n = 58 rather than n = 190 was considered sufficient given the circumstances and the fact that the analysis revealed acceptable reliability. Lastly, it should be noted that the data consisted of low levels of missing item responses (n = 9), which could indicate that the p-CECSS-S is easy to use.

## Conclusions

Our translation of the CECSS and testing of the final version supports that the p-CECSS-S is a user-friendly, three-dimension questionnaire with high internal consistency and stability that screens for pediatric emergency care satisfaction in a Swedish setting. Hence, it is our recommendation that future research address its psychometric properties across more diverse pediatric populations and healthcare settings.

## Data Availability

The datasets generated and/or analyzed during the current study are not publicly available for ethical reasons.

## Abbreviations

CECSS: Consumer Emergency Care Satisfaction Scale
p-CECSS-S: Swedish Pediatric Consumer Emergency Care Satisfaction Scale
ED: Emergency department
ICC: Intraclass correlation coefficient
I-CVI: Item-content validity index
KIDSCREEN-52: Health-Related Quality of Life Questionnaire for Children and Adolescents Aged from 8 to 18 years
KIDSCREEN-37: Health-Related Quality of Life Questionnaire for Children Aged from 4 to 7 years
KMO: Kaiser–Meyer–Olkin measure
n: Sample size
N: Population size
PN: Pediatric nurse
RETTS-p: Rapid Emergency Triage and Treatment System Pediatrics
S-CVI: Scale-content validity index
SD: Standard deviation
SPSS: Statistical Package for the Social Sciences
